# A Critic Criticised

**Published:** 1891-02-07

**Authors:** 


### Passing Topics.

### A CRITIC CRITICISED.

Mr. Andrew Tuer, whose name is perhaps best known to managers of charities in connection with his sweeping denunciation of charitable appeals as so many "lithographed lies," has recently published a letter in which those who think they have suffered at his hands will not be slow to detect the explanation of his unsympathetic bearing. The numerous communications with which this gentleman favours the editors of our journals supply in themselves some evidence of a restless habit, but we owe it to his own frank utterance that we are now aware of his irascibility under trivial provocation, and are at the same time afforded indications of the way to render an appeal less obnoxious.

"I confess," writes Mr. Tuer to the Editor of the *Times*, upon the momentous subject of uncut books; "I confess to a feeling of pleasure in slowly opening out and turning over the leaves of a beautiful book, but if I jagged its edges and littered the floor with odd bits irascibly twitched out, I should entrust the operation to a person of more placid temperament." This appears to us a fitting and pregnant pronouncement. True, the subject is not profound or weighty or engrossing, unless to a bibliophile, but it is from this very circumstance that we cull our first lesson and remark how great a part trifles play in the history of the world. We perceive, too, that to the truly fastidious the matter is as nothing compared with the way in which it is presented, and inferentially we gather that even "lithographed lies" might be acceptable if only the lithography were perfect.

We fear we must admit that certain collectors have already discovered this for themselves, and hence the superfine and artistic publications many of them affect. In so far as Mr. Tuer is impatient of the exaggeration and *suggestio falsi* so often associated with appeals, we are at one with him. But why this indiscriminating generalisation, this denouncing of good and bad alike? And, further, what is the substitute for appeals? We have asked this before, but we have never succeeded in getting a reply. This is an age of advertising, and why should charities which work for the general weal be denied a means of adding to their usefulness which is freely open to every tradesman desirous of advancing his individual interests?

Even the delightful publications Mr. Tuer's firm ushers into the world would not find so ready a sale if they were not judiciously pushed and, may we add, puffed. We lately received two circulars in one day about a book issued from the Leadenhall Press. Did we therefore write to the papers to denounce the suggestion to subscribe as pestiferous persecution? On the contrary, we have every intention of buying the book, and feel quite sure a treat awaits us. Apropos of this subject, we observe that a minor, but very deserving hospital, is now publicly making known these two facts: (1) That in order to obtain the means to treat the poor little sick children brought to its doors, it must mortgage its land and buildings; and (2) that when a general meeting was called to give the necessary powers, there was not enough interest among its constituents to allow of the formation of a quorum, and the meeting had to be adjourned accordingly. Here, at least, are no lithographed lies; unhappily, they are stereotyped truths. Want of money and paucity of interest are the twin troubles of most of our hospitals, and neither the one nor the other is likely to be remedied without persistent pleading. It is said that everything comes to him who waits. Hospitals have been waiting these many years for a more generous appreciation and support. It will be unfortunate for all parties if, in the end, they must carry their appeals to the parish, and become dependent upon rates. Agricola.

